# Molecular Science for Drug Development and Biomedicine

**DOI:** 10.3390/ijms151120072

**Published:** 2014-11-04

**Authors:** Wei-Zhu Zhong, Shu-Feng Zhou

**Affiliations:** 1 Gordon Life Science Institute, Belmont, MA 02478, USA; 2 Department of Pharmaceutical Sciences, College of Pharmacy, University of South Florida, Tampa, FL 33620, USA; E-Mail: szhou@health.usf.edu

With the avalanche of biological sequences generated in the postgenomic age, molecular science is facing an unprecedented challenge, *i.e.*, how to timely utilize the huge amount of data to benefit human beings. Stimulated by such a challenge, a rapid development has taken place in molecular science, particularly in the areas associated with drug development and biomedicine, both experimental and theoretical. The current thematic issue was launched with the focus on the topic of “Molecular Science for Drug Development and Biomedicine”, in hopes to further stimulate more useful techniques and findings from various approaches of molecular science for drug development and biomedicine.

The papers collected in this monograph can be categorized into the following four topics: (1) Pseudo Amino Acid Composition/Pseudo Oligonucleotide Composition; (2) Structure-Based Drug Design; (3) *In Vitro*/*In Vivo* Pharmacological Models; (4) Computational Model for Drug Development.

## 1. Pseudo Amino Acid Composition and Pseudo Oligonucleotide Composition

One of the most challenging problems in computational biology and biomedicine is how to formulate a biological sequence with a discrete model or a vector, yet still keep considerable sequence order information. To avoid completely losing the sequence-order information for proteins, particularly for their long-range or global sequence-order effects, the pseudo amino acid composition [1,2] or Chou’s PseAAC [3] was proposed. Ever since the concept of PseAAC was proposed in 2001, it has rapidly penetrated into almost all the areas of computational proteomics. Because it has been widely and increasingly used, in the paper entitled “PseAAC-General: Fast Building Various Modes of General Form of Chou’s Pseudo-Amino Acid Composition for Large-Scale Protein Datasets” [4], Professor Dr. Pufeng Du and his coworkers proposed a powerful software called “PseAAC-General” that can be used for fast building various modes of general form of Chou’s PseAAC for large-scale protein datasets, including the GO (Gene Ontology) mode, FunD (Functional Domain) mode, PSSM (Position-Specific Scoring Matrix) mode and many others as defined by the users according to their own desires and needs.

SNO (*S*-nitrosylation) is one of the most important and universal PTMs (posttranslational modifications) responsible for sensing and transducing signals to regulate various cellular functions and signaling events. In the article entitled “PSNO: Predicting Cysteine *S*-Nitrosylation Sites by Incorporating Various Sequence-Derived Features into the General Form of Chou’s PseAAC” [5], Dr. Zhiqiang Ma and coworkers developed a new bioinformatics tool to identify SNO sites in protein sequences by incorporating various sequence-derived features into the general form of Chou’s PseAAC, and achieved very promising results.

Encouraged by the successes of using PseAAC to deal with protein/peptide sequences, a question has naturally and logically occurred: how to use the similar approach to deal with DNA/RNA sequences? To address this problem, recently the pseudo oligonucleotide composition or PseKNC [6] and PseKNC-General [7] were developed. In the article with the title of “iRSpot-TNCPseAAC: Identify Recombination Spots with Trinucleotide Composition and Pseudo Amino Acid Components” [8], Dr. Wang-Ren Qiu and coauthors proposed a different approach to deal with this problem. They first convert a DNA sequence into a protein sequence by using the 3→1 rule from a 3-nucleotide codon to an amino acid, followed by using the Chou’s PseAAC to predict the recombination spots of DNA. Meanwhile, a publically accessible web-server for the prediction method has been established. Furthermore, for the convenience of the vast majority of experimental scientists, a step-by-step guide is also given on how to use the web server to obtain the desired result.

## 2. Structure-Based Drug Design

The article entitled with “Structure of *N*-Terminal Sequence Asp-Ala-Glu-Phe-Arg-His-Asp-Ser of Aβ-Peptide with Phospholipase A_2_ from Venom of Andaman Cobra Sub-Species *Naja naja sagittifera* at 2.0 Å Resolution” authored by Professor Dr. Zeenat Mirza, and coauthors reported the structure from Alzheimer’s Aβ-peptide in complex with phospholipase A_2_, which was determined by X-ray crystallography at 2.0 Å resolution [9]. Their findings suggest the possibility of interactions between *N*-terminus residues (DAEFRHDS) and phospholipase A_2_. Their study is a key step towards understanding the mechanism behind the Aβ and PLA_2_ interaction that may facilitate the development of novel therapeutic strategies against the inflammatory responses to retard many diseases.

Mostafa M. Ghorab and colleagues, in the article “Synthesis, Characterization and Anti-Breast Cancer Activity of New 4-Aminoantipyrine-Based Heterocycles” [10], reported that a new series of heterocycles synthesized by incorporating antipyrine moiety. They observed that these molecules have anticancer activity against human tumor breast cell line (MCF7). In their study, the authors utilized 4-aminoantipyrine as key intermediate for the synthesis of pyrazolone derivatives bearing biologically active moieties. As claimed by the authors, these findings might be of use for developing more potent and selective anti-breast cancer agents.

In their article “The Discovery of Potentially Selective Human Neuronal Nitric Oxide Synthase (nNOS) Inhibitors: A Combination of Pharmacophore Modelling, CoMFA, Virtual Screening and Molecular Docking Studies” [11], Dr. Guanhong Xu *et al.* presented a workflow for the identification and prioritization of compounds as potentially selective human nNOS inhibitors utilizing a three-dimensional pharmacophore model. They found that the identified hit compounds were structurally different from available inhibitors and may serve as potential leads or starting points for structural optimization to identify novel nNOS inhibitors.

As described in the paper entitled “Synthesis, Preliminary Bioevaluation and Computational Analysis of Caffeic Acid Analogues” [12], Dr. Weidong Zhang and coworkers designed, synthesized and evaluated a series of caffeic acid amides for the anti-inflammatory activity. They developed a 3D pharmacophore model on the basis of biological results for further structural optimization and also performed the predication of the potential targets using the PharmMapper server. Results from their study suggest that these amide analogues represent a promising class of anti-inflammatory scaffold for further exploration and target identification.

As reported in the article “Synthesis and Antioxidant Activity Evaluation of New Compounds from Hydrazinecarbothioamide and 1,2,4-Triazole Class Containing Diarylsulfone and 2,4-Difluorophenyl Moieties” [13], Dr. Stefania-Felicia Barbuceanu and coauthors synthesized the new hydrazinecarbothioamides, 1,2,4-triazole-3-thiones and *S*-alkylated 1,2,4-triazole derivatives, which were then characterized by IR, ^1^H-NMR, ^13^C-NMR and mass spectral data. The results obtained by them with the preliminary screening of antioxidant activity suggest that the molecules from hydrazinecarbothioamide class might serve as interesting compounds for the development of new antioxidant agents by synthesis of some new derivatives with this structure.

## 3. *In Vitro*/*In Vivo* Pharmacological Models

According to the report by Dr. Hong Jiang and coworkers in “Perineural Dexmedetomidine Attenuates Inflammation in Rat Sciatic Nerve via the NF-κB Pathway” [14], they have established a rat model that simulates a clinical surgical procedure to investigate the anti-inflammatory effect of perineural administration of dexmedetomidine and the underlying mechanism. Results from their studies suggest that dexmedetomidine inhibits the nuclear translocation and binding activity of activated NF-κB, thus reducing inflammatory cytokines. It may hold high potential for applying the dexmedetomidine as an adjuvant in peripheral nerve anesthesia.

Dr. Jin Yeul Ma and colleagues evaluated the inhibitory effects of palmultang (PM) on the production of inflammatory factors and on the activation of mechanisms in murine macrophages. They found that PM suppressed the expression of nitric oxide, inflammatory cytokines and inflammatory proteins by inhibiting nuclear factor (NF)-κB and mitogen-activated protein kinase (MAPK) signaling pathways and by inducing heme oxygenase (HO)-1 expression. Their results as detailed in the research article “Inhibitory Effects of Palmultang on Inflammatory Mediator Production Related to Suppression of NF-κB and MAPK Pathways and Induction of HO-1 Expression in Macrophages” suggest that PM could be developed as a new anti-inflammatory agent derived from natural products [15].

In the review paper entitled “Colonization and Infection of the Skin by *S. aureus*: Immune System Evasion and the Response to Cationic Antimicrobial Peptides” [16], Professor Dr. Yoonkyung Park and coworkers discussed the peptides (defensins, cathelicidins, RNase7, dermcidin) and other mediators (toll-like receptor, IL-1 and IL-17) that comprise the host defense against *S. aureus* skin infection, as well as the various mechanisms by which *S. aureus* evades host defenses. They anticipate that targeted drug development around highly conserved bacterial resistance mechanisms against host cationic antimicrobial peptides will be a promising pharmacologic approach in this era of highly virulent and drug-resistant strains of *S. aureus*.

Diallyl disulfide (DADS) is a natural organosulfur compound isolated from garlic. The anticancer mechanisms of DADS in human esophageal carcinoma have not been elucidated, especially *in vivo*. In the research article entitled “DADS Suppresses Human Esophageal Xenograft Tumors through RAF/MEK/ERK and Mitochondria-Dependent Pathways” contributed by Dr. Hongbing Ma and his colleagues [17], the authors reported that the DADS suppresses esophageal tumors without any apparent signs of toxicity, which is in agreement with a strong increase of apoptosis both *in vitro* and *in vivo*. They claimed that DADS might be a potentially effective and safe anti-cancer agent for esophageal carcinoma treatment.

In the article “4-Hydroxyphenylacetic Acid Attenuated Inflammation and Edema via Suppressing HIF-1α in Seawater Aspiration-Induced Lung Injury in Rats” [18], Drs. Xiaobo Wang and Faguang Jin, and coworkers conducted an investigation in the effect of 4-Hydroxyphenylacetic acid (4-HPA) on seawater aspiration-induced lung injury using a seawater drowning rat model *in vivo* and the hypoxia-inducible factor-1α (HIF-1α) siRNA and permeability assay *in vitro.* Their results indicated that 4-HPA attenuated inflammation and edema through suppressing hypertonic and hypoxic induction of HIF-1α in seawater aspiration-induced lung injury in rats, and hence may be considered as a potential agent in the treatment of seawater aspiration-induced lung injury.

Wound healing plays an important role in protecting the human body from external infection. Cell migration and proliferation of keratinocytes and dermal fibroblasts are essential for proper wound healing. In the research article entitled “Effects of the Novel Compound DK223 ([1*E*,2*E*-1,2-Bis(6-methoxy-2*H*-chromen-3-yl)methylene]hydrazine) on Migration and Proliferation of Human Keratinocytes and Primary Dermal Fibroblasts” [19], Dr. Moonjae Cho and colleagues identified a novel compound DK223 ([1*E*,2*E*-1,2-bis(6-methoxy-2*H*-chromen-3-yl)methylene]hydrazine) that concomitantly induced human keratinocyte migration and dermal fibroblast proliferation. They also found that DK223 simultaneously induced both keratinocyte migration via reactive oxygen species production and fibroblast proliferation via TGF-β1 induction.

## 4. Computational Model for Drug Development

With the huge amount uncharacterized proteins entering into the protein database, it is time-consuming and expensive to identify the protein-protein interactions (PPIs) by experiments alone. Therefore, it is highly demanding to develop computational methods for predicting PPIs. In the research article entitled “Prediction of Protein–Protein Interaction with Pairwise Kernel Support Vector Machine” [20], Professor Dr. Shaowu Zhang and his colleagues offered a novel method along with its web-server PPI-PKSVM developed by using the two feature extraction approaches (DFPCA and AAID) to represent the protein sequence samples, followed by using the pairwise kernel function support vector machine model. They conclude that the predicted results are very encouraging and promising for predicting PPIs according to the sequence information alone.

Nuclear receptors (NRs) are closely associated with various major diseases such as cancer, diabetes, inflammatory disease, and osteoporosis. Therefore, NRs have become a frequent target for drug development. During the process of developing drugs against these diseases by targeting NRs, we are often facing a problem: Given a NR and chemical compound, can we identify whether they are really in interaction with each other in a cell? To address this problem, in the article “iNR-Drug: Predicting the Interaction of Drugs with Nuclear Receptors in Cellular Networking” [21], Dr. Xuan Xiao *et al.* proposed a predictor called “iNR-Drug” in which the drug compound concerned was formulated by a 256-D (dimensional) vector derived from its molecular fingerprint, and the NR a 500-D vector formed by incorporating its sequential evolution information and physicochemical features into the general form of Chou’s PseAAC. Compared with the existing prediction methods in this regard, iNR-Drug not only can yield a higher success rate, but is also featured by a user-friendly web-server, which is particularly useful for most experimental scientists to obtain their desired data in a timely manner.

Inherently chiral calix[4]arenes can be theoretically regarded as a type of complex planar chiral molecule when bridging carbons are treated as achiral and each phenyl ring and its six substituents treated as coplanar. Based on one approximation and one hypothesis, Drs. Shao-Yong Li, Wei Qiao and Jun-Min Liu, and their colleagues have derived a expression for qualitatively analyzing the microhelical electronic energy, as elaborated in the article “Qualitative Analysis of the Helical Electronic Energy of Inherently Chiral Calix[4]arenes: An Approach to Effectively Assign Their Absolute Configuration” [22]. According to their report, the scientificity and effectiveness in absolute configuration assignments of inherently chiral calix[4]arenes were almost entirely confirmed for all of the entities whose absolute configurations and optical rotation signs have been ascertained.

It is a great challenge to elucidate the polypharmacological mechanisms of polyphenols. In the research article “Elucidating Polypharmacological Mechanisms of Polyphenols by Gene Module Profile Analysis” [23], Dr. Hong-Yu Zhang and coworkers have developed a method for identifying the multiple targets of chemical agents through analyzing the module profiles of gene expression upon chemical treatments. By using this method, they have identified 148 targets for 20 polyphenols derived from cMap. As claimed by these authors, a large part of the targets were validated by experimental observations, implying that the medicinal effects of polyphenols are far beyond their well-known antioxidant activities.

In the last decade or so, it has been observed that many molecular biosystems and biomedical systems belong to the multi-label systems where each of their constituent molecules may possess two or more attributes, functions or features, and hence need multiple-label or multi-target method to analyze them [24]. In the paper entitled “Prediction of Multi-Target Networks of Neuroprotective Compounds with Entropy Indices and Synthesis, Assay, and Theoretical Study of New Asymmetric 1,2-Rasagiline Carbamates” [25], Professor Drs. Humberto González-Díaz and Xerardo García-Mera and their colleagues used Shannon entropy measures to develop predictive models for multi-target networks of neuroprotective/neurotoxic compounds. Their method has been demonstrated to be a useful complementary tool in the organic synthesis and evaluation of the multi-target biological activity of new compounds with potential neuroprotective activity, as well as in the prediction of complex networks of drug-target interactions.

As one can see from the aforementioned nineteen papers collected in this special issue they are all featured by either developing powerful tools or reporting important findings, which will be very useful for both the basic research in molecular sciences and drug design in pharmaceutical industry.

It is our hope that publication of this special issue can stimulate more powerful tools in computational biomedicine as well as more profound findings in treating diseases so as to benefit human beings.
